# The personality traits with depression and suicidal ideation among Thai medical students: a university-based multiregional study

**DOI:** 10.1186/s40359-024-01707-8

**Published:** 2024-04-23

**Authors:** Jarurin Pitanupong, Adchara Sa-i, Katti Sathaporn, Aimorn Jiraphan, Pichai Ittasakul, Nuntaporn Karawekpanyawong

**Affiliations:** 1 Department of Psychiatry, Faculty of Medicine, Prince of Songkla University, Hat Yai, Songkhla, 90110 Thailand; 2 Department of Psychiatry, Faculty of Medicine, Ramathibodi Hospital, Mahidol University, Bangkok, Thailand; 3 Department of Psychiatry, Faculty of Medicine, Chiang Mai University, Chiang Mai, Thailand

**Keywords:** Coping mechanisms, Depression, Medical student, Personality traits

## Abstract

**Background:**

The prevalence of depression in medical students was greater than in the general population. Knowing of predictive factors for depression among medical students is useful. The objectives of this study included the assessment of personality traits as well as the association between the personality traits and the presence of symptoms of depression, and suicidal ideation among medical students covering several regions of Thailand.

**Methods:**

From April to July 2023, a cross-section study was conducted. The participants included first to sixth-year medical students studying at three Faculties of Medicine in Thailand; Prince of Songkla University; Ramathibodi Hospital, Mahidol University; and Chiang Mai University. Using the online process, the questionnaires were composed of three sections; demographic data; the International Personality Item Pool-NEO (IPIP-NEO), Thai version; and the Patient Health Questionnaire-9 (PHQ-9), Thai version. Demographics, personality traits, depression, and suicidal ideation were analyzed using descriptive statistics. The results were presented as frequency, mean, and standard deviation (SD) or median and interquartile range (IQR). The association between independent variables and the presence of depression was identified using binary logistic regression analysis, and the association with suicidal ideation was identified using ordinal logistic regression analysis.

**Results:**

The 868 medical students participated in this study. Most of them were female (63.5%), Buddhist (82.0%), and first-year medical students (31.8%). The mean age (SD) was 20.8 (2.2) years, and the mean cumulative Grade Point Average (SD) was 3.5 (0.4). They reported the median (IQR) score of PHQ-9 as 6.0 (3.0–9.0), 238 participants (27.4%) presented with depression, and 138 (15.9%) participants reported suicidal ideation. According to the IPIP-NEO, participants with depression or suicidal ideation had higher Neuroticism scores and lower Extraversion, Agreeableness, and Conscientiousness scores compared to those without such issues. An increase in the Neuroticism score was linked to higher odds of depression, while an increase in the Conscientiousness score was associated with lower odds of depression. Suicidal ideation significantly increased with higher Neuroticism scores and the presence of a psychiatric illness.

**Conclusions:**

More than a quarter of Thai medical students reported depression. A higher Neuroticism and lower Extraversion, Agreeableness, and Conscientiousness scores related to depression. Therefore, medical schools may benefit from knowing medical students’ personality traits, to identify coping mechanisms and predict those at a higher risk of developing depression in the future.

**Supplementary Information:**

The online version contains supplementary material available at 10.1186/s40359-024-01707-8.

## Background

Major depressive disorder (MDD) is one of the most common psychiatric disorders, with a prevalence of 4.7% [1]. Among medical students, the prevalence of MDD is higher than that of the general population; ranging from 20.7–70.8% [2, 3]. Among Thai medical students, studies have identified high stress levels (38.6%) [4], and poor mental health (29.6–32.1%) [5, 6]. Two-thirds (62.8%) of sixth-year medical students showed a high level of burnout [6]. In 2017–2019, a study reported the prevalence of MDD among medical students in Central [7, 8], Northern [9], Northeast [10], and Southern [11] Thailand which ranged from 4.9% to 25.8%.

Suicidal ideation (SI) was frequent and suicide was one of the leading causes of death among medical students. A prior study reported the prevalence of SI among medical students in Brazil was 7.2% [12]. Among medical students in Northern Thailand, the study reported the prevalence of SI was 12.5% [9]. The associated factors with SI were symptoms of MDD, and living alone [12]. However, several protective factors of MDD among medical students included: having mindful thoughts or a growth mindset, having their own decision to study at medical school [7, 8], having a positive family relationship [8], having a personal counselor or good social support, and adequate mentoring programs [7]. However, personal vulnerabilities, intrinsic motivation, disinterest in medical studies, parents’ decisions to study medicine, academic achievement [10], and learning environments [7, 10] were the factors related to MDD. In particular, lack of social support and having relationship problems were mentioned as associated with more severe and persistent symptoms of MDD [7].

Concerning personality traits, the NEO Personality Inventory (NEO-PI), or Five-Factor Model (FFM), is a measurement test for personality traits. It divides personality aspects into five domains; including Neuroticism (N) (sensitive/nervous), Extraversion (E) (outgoing/energetic), Openness (O) (inventive/curious), Agreeableness (A) (friendly/compassionate), and Conscientiousness (C) (efficient/organized) [13, 14]. Using the NEO-PI, many studies have reported that individuals with MDD showed a higher score of Neuroticism [15–18], a lower score of Extraversion [15], and Conscientiousness [18]. A higher score of Neuroticism was associated with greater odds of suicide attempts [17] and the presence of depressive residual symptoms, despite receiving antidepressants [18–20].

Because the rate of mental health problems, burnout, and MDD among Thai medical students is higher than in the general population [2, 3]. There is a tendency that the rate will be higher in the future [11]. Therefore, modifiable issues related to the predictive factors of MDD should be highlighted. Personality traits by their nature may be persistent, but actually, they can change by experience over a lifetime. More and proper coping styles can be learned [17]. In Thailand, there has never been a study on personality traits with MDD among Thai medical students before. Thus, this study aimed to find out the personality traits as well as the association between the personality traits and the presence of symptoms for MDD, and SI among medical students covering several regions; including Northern, Central, and Southern Thailand. For the faculties, the result will provide valuable data to identify problematic personality and coping mechanisms in medical students who are more prone to develop MDD.

## Methods

After being approved by the three corresponding ethics committees, from the Faculty of Medicine, Prince of Songkla University (REC: 65–488-3–1), the Faculty of Medicine, Ramathibodi Hospital, Mahidol University (REC: MURA 2023/353), and the Faculty of Medicine, Chiang Mai University (REC: PSY-2566–09439), this cross-sectional study was conducted on all medical students attending these three Faculties of Medicine; Prince of Songkla University; Ramathibodi Hospital, Mahidol University; and Chiang Mai University. From April to July 2023, the first to sixth-year medical students of the above-mentioned institutions were surveyed by an online process. All medical students who were members of social media, whose age was 18 years or more, who used the Thai language; who agreed to participate in the study, and who were willing to complete all of the questionnaires were included. Meanwhile, those who were unable to complete the questionnaire, or declined and decided to withdraw from the study were excluded.

The sample size calculation was based on the literature review and found that the mean scores (SD) for the Neuroticism (N) and Extraversion (E) personality traits in connection to the general population were 50.8 (10.6) and 53.3 (47.5), respectively [15]. Therefore, the calculated sample size, using the estimation of one sample mean formula; given alpha = 0.05 and precision less than 10.0% of the mean scores, would need to include at least 347 medical students. However, from previous data collection via the online platform, there was approximately 60.0% non-response rate [21]. Therefore, the revised sample size was: n (new) = n / 1- non-response rate, which was n (new) = 347/0.4 = 868.

The data collection was conducted by convenience process, the researcher used personal contacts to request permission and convey information regarding the study to the admin of the medical student's official social media. All participants were provided with the rationale, and overview of the research by the researcher, so they could consider whether to collaborate in the study or not. Participants were invited to collaborate in the study by either clicking the provided link or scanning a QR code through social media advertisements to access the online questionnaire. All participants were allowed to finish and submit the questionnaires immediately or at a later time using the online process. They retained the right to withdraw from the research at any time without giving any reason. Adhering to the policy of strict confidentiality, the signatures of the respondents were not required, and the data were stored in a secure place. Only the researcher could access the information via a password.

### Questionnaires

1. General demographic information included age, sex, religion, income, medical school, academic year, cumulative Grade Point Average (GPA), physical and psychiatric illness, and history of alcohol and substance usage.
2. To assess personality traits, the International Personality Item Pool-NEO (IPIP-NEO) Thai version, was used. This tool is a self-report questionnaire evaluating an individual’s character using thirty questions divided into five domains; Neuroticism (N); Extraversion (E); Agreeableness (A); Openness (O); and Conscientiousness (C). Each question would be answered on a 5-point rating scale; 1 (disagree strongly or very inaccurate); 2 (disagree a little or moderately inaccurate); 3 (neither agree nor disagree, or neither accurate nor inaccurate); 4 (agree with a little or moderately accurate); 5 (strongly agree or very accurate). This questionnaire was tested for internal consistency with a Cronbach's alpha coefficient of *N* = 0.83, E = 0.76, O = 0.67, A = 0.37, and C = 0.73 [22]. For the data in this study, the IPIP-NEO questionnaire demonstrated internal consistency with a Cronbach's alpha coefficient of 0.85; *N* = 0.83, E = 0.80, O = 0.62, A = 0.79, and C = 0.76.
3. To assess depressive symptoms, the Patient Health Questionnaire-9 (PHQ-9) Thai version was chosen, because it is the standard screening tool to detect MDD being used in Thai clinical practice. PHQ-9 is a self-rating questionnaire that consists of nine questions. Each question would be answered on a 4-point rating scale; 0 (never); 1 (rarely); 2 (sometimes); 3 (always). The total score was interpreted according to; 0–4 (no or minimal depression); 5–9 (mild depression); 10–14 (moderate depression); 15–19 (moderately severe); 20–27 (severe depression). Item 9 of the PHQ-9 was used to evaluate suicidal ideation. This questionnaire was tested for internal consistency and found a Cronbach's alpha coefficient of 0.79, a sensitivity of 0.53, and a specificity of 0.98. It has acceptable psychometric properties for the screening of MDD in general practice, with a recommended cut-off score of nine or greater [23]. The PHQ-9 questionnaire demonstrated internal consistency with a Cronbach's alpha coefficient of 0.85 for the data in this study.

### Statistical analysis

Descriptive statistics were calculated for demographic data (including sex, religion, academic year, GPA, physical and psychiatric illness, alcohol and substance usage), personality traits, depression, and SI. The result was presented as frequency, mean, and standard deviation (SD) or median and interquartile range (IQR) if the data was not normally distributed. The analysis of the association between independent variables and the presence of MDD was identified using Chi-square or Fisher’s exact tests, Wilcoxon rank sum test, and binary logistic regression analysis. The association between independent variables and SI was identified using the Kruskal–Wallis test and ordinal logistic regression analysis. We selected the variables that had *p*-value < 0.2 in the univariate analysis as inputs for multivariate analysis using backward elimination. To address potential confounding, we carefully identified and considered potential confounders based on both existing literature and the variables that the researcher was interested in. The likelihood ratio (LR) test was conducted to assess the model fit. The *p*-value for significance in the LR test was set at 0.05.

## Results

### Demographic characteristics

Of 1,224 first to sixth-year medical students, 868 participated in this study: 717 from Prince of Songkla University, 109 from Chiang Mai University, and 42 from Mahidol University. The response rate was 70.9%. The participants had a mean age (SD) of 20.8 (2.2) years. Most of them were female (63.5%), Buddhist (82.0%), and first-year medical students (31.8%), with a mean cumulative GPA (SD) of 3.5 (0.4). Mostly, they reported no previous physical illness (83.8%), or psychiatric illness (91.5%). The most common psychiatric illness was MDD (3.2%), generalized anxiety disorder (GAD) (1.4%), adjustment disorder (1.4%), and dysthymia (0.7%) (Table 1).

**Table 1:** Demographic characteristics (*N* = 868)

Demographic characteristics	Number (%)
**Sex**	
Female	551 (63.5)
Male	317 (36.5)
**Religion**	
Buddhism	712 (82.0)
Islam	95 (10.9)
Christianity/others	57 (6.6)
No answer	4 (0.5)
**Academic year**	
1st year	276 (31.8)
2nd year	139 (16.0)
3rd year	73 (8.4)
4th year	151 (17.4)
5th year	120 (13.8)
6th year	109 (12.6)
**Physical illness**	
No	727 (83.8)
Yes	141 (16.2)
**Income (**Baht)	
< 5000	163 (18.8)
5000–10000	482 (55.5)
10,001–20000	172 (19.8)
> 20,000	21 (2.4)
No answer	30 (3.5)
**Psychiatric illness**	
No	794 (91.5)
Yes	74 (8.5)
MDD / Dysthymia	34 (3.9)
GAD / Insomnia	14 (1.6)
Adjustment disorder	12 (1.4)
Attention deficit hyperactivity disorder	6 (0.7)
Bipolar disorder	2 (0.2)
Not specify	6 (0.7)
**Alcohol usage**	
No	856 (98.6)
Yes	9 (1.0)
No answer	3 (0.3)
**Substance usage**	
No	856 (98.6)
Yes	6 (0.7)
No answer	6 (0.7)

### Depressive symptom profiles

**Fig. 1:**
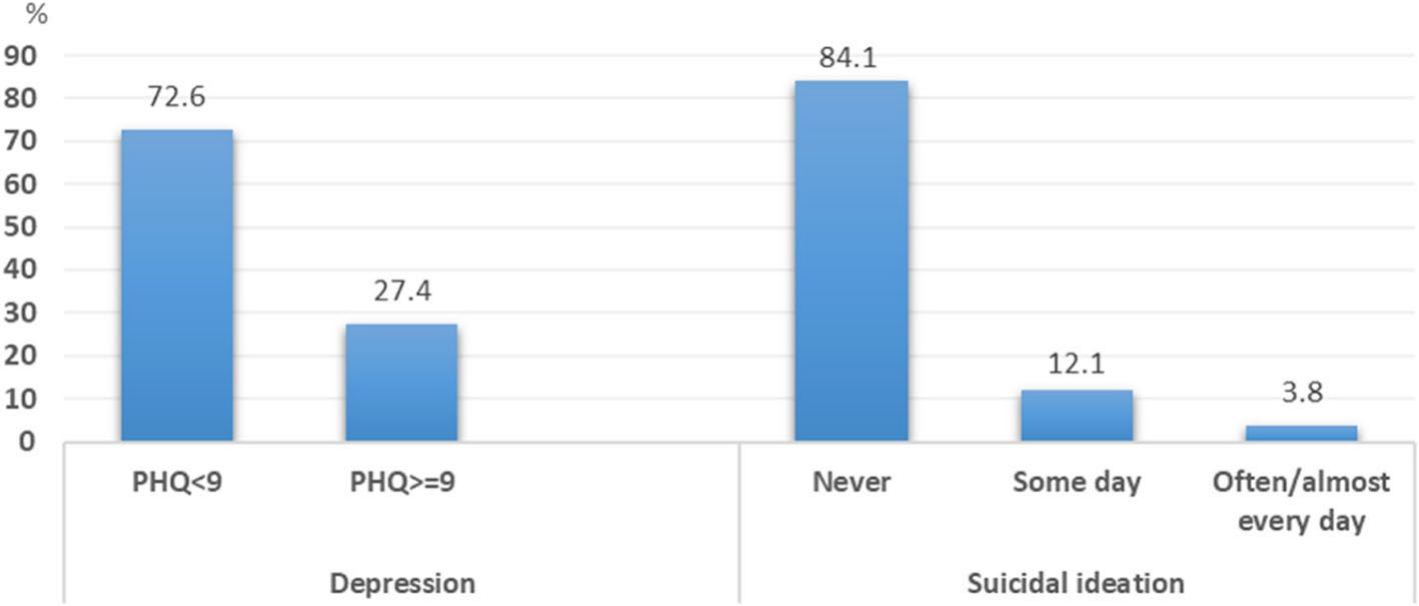
Depression and Suicidal ideation (*N* = 868)

**Fig. 2:**
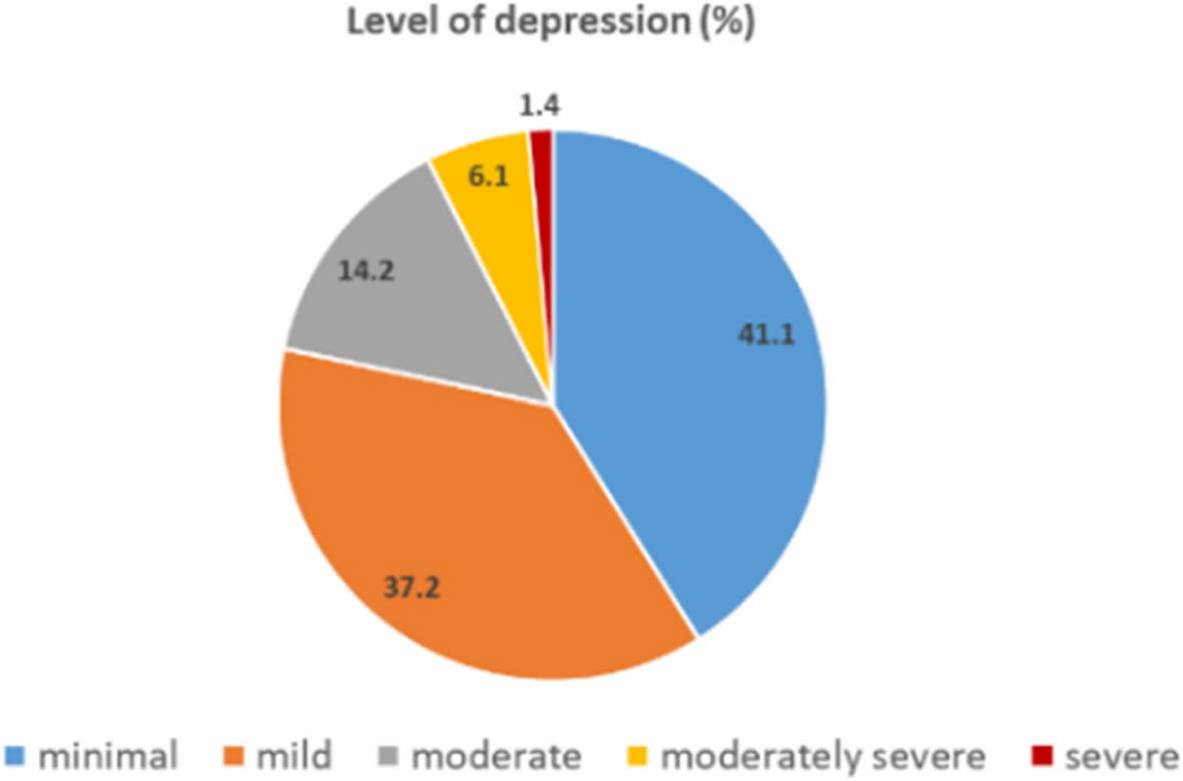
Level of depression (*N* = 868)

**Fig. 3:**
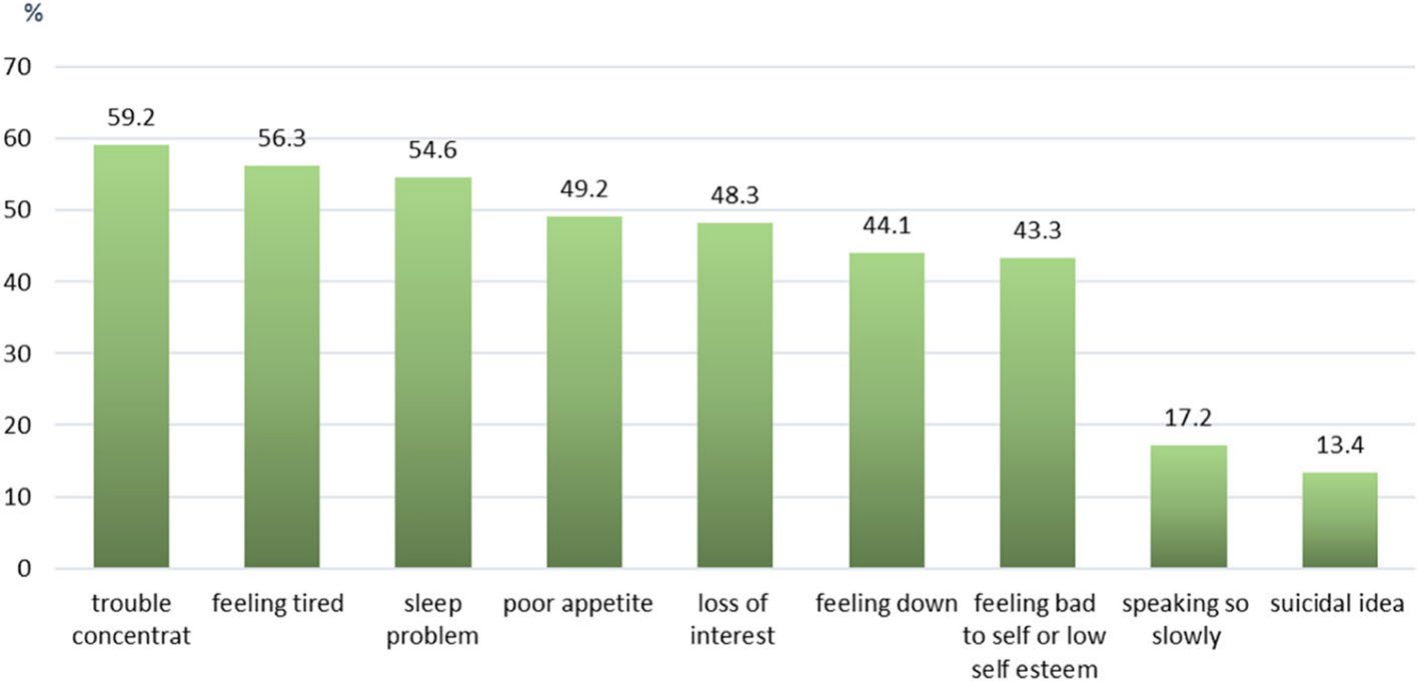
The symptoms of depression (*N* = 238)

Of all 868 participants, the median (IQR) score of PHQ-9 was 6.0 (3.0–9.0). And 238 participants (27.4%) had a PHQ-9 score of nine or greater, which indicated the presence of MDD (Fig. 1). The level of depression is shown in Fig. 2. The common symptoms of MDD were; trouble with concentration (59.2%), feeling tired (56.3%), sleep problems (54.6%), and poor appetite (49.2%) (Fig. 3). Additionally, 138 (15.9%) participants reported SI (Figs. 1, and 3). Moreover, among participants who had a history of MDD and dysthymia, 22 participants (64.7%) reported having a PHQ-9 score of nine or greater.

There was a statistically significant association between GPA and the presence of a history of psychiatric illness with the presence of MDD. For SI, academic year, GPA, the presence of a history of physical illness, and psychiatric illness were statistically significantly associated variables in univariate analysis (Table 2).

**Table 2:** Association of demographic profile and the presence of depression or suicidal ideation: univariate analysis (*N* = 868)

**Variables**	**Presence of depression**			***P*-value**	**Presence of suicidal ideation**				***P*-value**
	**PHQ < 9** (N = 630)	**PHQ ≥ 9** (N = 238)	**Chi2***(df)*		**Never** (N = 730)	**Some day** (N = 105)	**Often / almost every day** (N = 33)	**Chi2***(df)*	
**Sex**			1.03*(1)*	0.310				2.58*(2)*	0.276
Male	237 (37.6)	80 (33.6)			273 (37.4)	31 (29.5)	13 (39.4)		
Female	393 (62.4)	158 (66.4)			457 (62.6)	74 (70.5)	20 (60.6)		
**Religion**			0.26*(2)*	0.879				Fisher’s exact test	0.433
Buddhism	515 (82.1)	197 (83.1)			595 (81.8)	90 (86.5)	27 (81.8)		
Islam	71 (11.3)	24 (10.1)			85 (11.7)	8 (7.7)	2 (6.1)		
Christianity/other	41 (6.5)	16 (6.8)			47 (6.5)	6 (5.8)	4 (12.1)		
**Academic year**			0.94*(1)*	0.333				6.59*(2)*	0.037
Pre-clinical	361 (57.3)	127 (53.4)			423 (57.9)	52 (49.5)	13 (39.4)		
Clinical	269 (42.7)	111 (46.6)			307 (42.1)	53 (50.5)	20 (60.6)		
**GPA** < 3.00	56 (8.9)	35 (14.7)	11.41*(3)*	0.009	64 (8.8)	20 (19)	7 (21.2)	24.77*(6)*	< 0.001
3.01–3.49	135 (21.4)	59 (24.8)			156 (21.4)	25 (23.8)	13 (39.4)		
> 3.5	342 (54.3)	102 (42.9)			390 (53.4)	43 (41)	11 (33.3)		
Not specify	97 (15.4)	42 (17.6)			120 (16.4)	17 (16.2)	2 (6.1)		
**Physical illness**			0.63*(1)*	0.428				9.54*(2)*	0.008
No	532 (84.4)	195 (81.9)			622 (85.2)	77 (73.3)	28 (84.8)		
Yes	98 (15.6)	43 (18.1)			108 (14.8)	28 (26.7)	5 (15.2)		
**Psychiatric illness**			30.32*(1)*	< 0.001				50.23*(2)*	< 0.001
No	597 (94.8)	197 (82.8)			689 (94.4)	79 (75.2)	26 (78.8)		
Yes	33 (5.2)	41 (17.2)			41 (5.6)	26 (24.8)	7 (21.2)		
**Alcohol use**			Fisher’s exact test	0.269				Fisher’s exact test	0.176
No	622 (99.2)	234 (98.3)			721 (99.2)	102(97.1)	33 (100)		
Yes	5 (0.8)	4 (1.7)			6 (0.8)	3 (2.9)	0 (0)		
**Substance use**			Fisher’s exact test	0.354				Fisher’s exact test	0.344
No	622 (99.5)	234 (98.7)			720 (99.4)	103 (98.1)	33 (100)		
Yes	3 (0.5)	3 (1.3)			4 (0.6)	2 (1.9)	0 (0)		

### Personality profiles of medical students

The result of this study found a statistically significant difference in almost every domain of personality, except for Openness, regarding the IPIP-NEO Thai version between the subjects who reported having MDD/SI and those who did not. The group of subjects with the presence of MDD/SI had a higher average score in the Neuroticism domain and a lower average score in the Extraversion, Agreeableness, and Conscientiousness domains (Tables 3 and 4).

**Table 3:** Personality traits and the presence of depression (*N* = 868)

**Personality traits**	**Total** (*N* = 868)	**Presence of depression**		**Ranksum-test *P*-value**
		**PHQ < 9** (*N* = 630)	**PHQ ≥ 9** (*N* = 238)	
**Neuroticism**				< 0.001^*^
Median (IQR)	18 (14,23)	16 (13,20)	24 (20.2,28)	
**Extraversion**				< 0.001^*^
Median (IQR)	10 (8,12)	10 (8,12)	9 (6.2,11)	
**Agreeableness**				< 0.001^*^
Median (IQR)	19 (17,21)	19 (17,22)	18 (16,20)	
**Openness**				0.762
Median (IQR)	23 (20,26)	23 (21,26)	24 (20,26)	
**Conscientiousness**				< 0.001^*^
Median (IQR)	28 (25,32)	30 (26,33)	25 (22,27.8)	

^*^Statistically significant difference between group

**Table 4:** Personality traits and the presence of suicidal ideation (*N* = 868)

Personality traits	Presence of suicidal ideation			Kruskal–Wallis test *P*-value
	**Never** (*N* = 730)	**Some day** (*N* = 105)	**Often/almost everyday** (*N* = 33)	
**Neuroticism**				< 0.001^*^
Median (IQR)	17 (14,21)	24 (21,28)	26 (24,29)	
**Extraversion**				< 0.001^*^
Median (IQR)	10 (8,12)	9 (6,11)	9 (6,10)	
**Agreeableness**				0.002^*^
Median (IQR)	19 (17,21)	18 (16,20)	18 (15,20)	
**Openness**				0.494
Median (IQR)	23 (21,26)	24 (20,26)	23 (18,26)	
**Conscientiousness**				< 0.001^*^
Median (IQR)	29 (25,32)	25 (22,28)	26 (24,29)	

^*^Statistically significant difference between group

### Association of personality profile, depression, and presence of suicidal idea

The final logistic regression model showed the association of personality profiles with MDD. Each increment of 1 point on the Neuroticism score was associated with an increase in the odds of MDD, with an odds ratio of 1.34 (95% CI = 1.28 to 1.41). On the contrary, the odds of MDD, with the ratio of 0.88 (95% CI = 0.84 to 0.92), was statistically significantly reduced for each increment of 1 point of the Conscientiousness score (Table 5).

**Table 5:** The final logistic regression model for the association with depression (*N* = 868)

**Factors**	**Crude OR** (95% CI)	**Adjusted OR** (95% CI)	***P*-value** (LR-test)
Neuroticism score	1.35 (1.29,1.41)	1.34 (1.28,1.41)	< 0.001
Agreeableness score	0.92 (0.89,0.96)	1.13 (1.07,1.2)	< 0.001
Conscientiousness score	0.82 (0.79,0.85)	0.88 (0.84,0.92)	< 0.001

The result from the ordinal logistic regression model showed the association of personality profiles with SI. The SI significantly increased with the Neuroticism score, and by having a psychiatric illness. One-point increment in Neuroticism score was associated with an increase in the odds of SI frequency according to the PHQ-9 rating scale (OR = 1.259, 95% CI = 1.211 to 1.312). Similarly, the odds of having someday and often/almost every day SI was increased by a factor of 1.946 if the participant had a psychiatric illness (Table 6).

**Table 6:** The final ordinal regression model for the association with suicidal ideation (*N* = 868)

Factors	Ordinal odds ratio	95% CI	*P*-value
Neuroticism score	1.259	1.211, 1.312	< 0.001
Having psychiatric illness	1.946	1.109, 3.357	0.018

## Discussion

From this study result, the prevalence of MDD and SI among Thai medical students was 27.4% and 15.9%, respectively. This research identified statistically significant differences in personality traits, regarding the traits of Neuroticism, Extraversion, Agreeableness, and Conscientiousness between the medical students with MDD/SI and those without MDD/SI. The medical students who reported MDD/SI had a higher score on Neuroticism, but a lower score on Extraversion, Agreeableness, and Conscientiousness domain. A higher Neuroticism score was associated with an increment in the odds of MDD. While the increment of the Conscientiousness score was associated with a reduction in the odds of MDD. SI significantly increased with the Neuroticism score and by having a psychiatric illness.

The prevalence of MDD among medical students in this study was in line with the prior meta-analysis, which reported a global prevalence of MDD among medical students of 28.0% [24]. This prevalence rate was still higher than the general population [1]. However, the prevalence of SI, identified in our study, was higher than before. Previously, the reported prevalence of SI among medical students in Brazil [12] and Northern Thailand [9] was 7.2% and 12.5%, respectively. Because SI was one of the leading causes of death among medical students [12], this finding raises our awareness that MDD and SI among medical students should deserve special attention. Moreover, our study found that 22 (64.7%) participants, who had a history of MDD or dysthymia, reported still having symptoms of MDD by PHQ-9 score. A possible explanation for this finding could be that they did not receive treatment, received inadequate treatment, or despite receiving treatment they had residual symptoms of MDD [25, 26]. Thus, this issue should be brought to the attention of medical schools and health authorities. All parties involved should offer early detection and set prevention programs, and interventions for MDD and SI among medical students before graduation.

Personality traits accessed by IPIP-NEO have been studied for a long time in patients with psychiatric illnesses. The most consistent finding was that traits of Neuroticism were higher in individuals with MDD [15, 18, 27–29]. The meta-analysis in 2010, including 175 studies published from 1980 to 2007, studied individuals with MDD, anxiety, and substance use disorders who reported low Conscientiousness scores and high Neuroticism scores in all diagnostic groups [27]. In 2013 Netherland study in older adults, identified that low Extraversion, and Conscientiousness scores and high Neuroticism scores were significantly associated with the presence and severity of MDD [28]. In a more detailed study in the USA in 2013, investigating the facets of each personality domain, the study reported that MDD was related to lower Extraversion (and facets of activeness, assertiveness, and positive emotionality) and Conscientiousness (and facets of competence, dutifulness, order, and self-discipline) and higher Neuroticism (and all its facets; anxiety, depressiveness, hostility, impulsivity, self-consciousness, and stress-vulnerability) [29]. The result of such personality profiles in individuals with MDD showed consistency across cultures. In a Japanese study, in 2013, individuals with treatment-resistant MDD showed a low score of Extraversion, Openness, and Conscientiousness, and a high score of Neuroticism [15].

In Thailand, 2023, regarding the IPIP-NEO Thai version, the study showed that individuals with MDD scored lower on the Extraversion and Conscientiousness domains, and higher on the Neuroticism domain [18]. Our study was done in a specific population of medical students. Similarly, the finding was congruent with the previous studies. We found statistical differences in personality traits between medical students with MDD and those without MDD. The medical students with MDD had significantly lower Extraversion and Conscientiousness scores and higher Neuroticism scores. Additionally, Openness domains were not associated with MDD. This finding was also similar to the prior study [28, 29]. As the Openness domain was related to inventiveness, and curiosity [20]. A possible explanation for this finding could be that the presence of MDD may not affect inventive, and curious behavior. Another explanation is the sample size in this study may not be enough to detect such an association.

Trying to identify the association between personality traits and SI, this study showed the same association reported in MDD. A possible explanation for this result could be that the symptoms of MDD and SI might be the same issue. In addition, a higher Neuroticism score and having a psychiatric illness were associated with SI. This is the same as the prior study that reported a higher score of Neuroticism was associated with greater odds of suicide attempts [17]. Therefore, the Neuroticism domain may be used as one predictor of risk for SI. Moreover, this study identified having a psychiatric illness was associated with SI. A possible explanation for this finding could be that half of these medical students were MDD/dysthymia and they may receive inadequate antidepressant treatment. This may cause the presence of residual symptoms; including SI. Therefore, among medical students with a history of psychiatric disorders, special attention should be given to thoughts of self-harm; as this is considered a risk to life.

The result from this study cannot be concluded in a cause-effect fashion due to the cross-sectional study design. In the prior study using Mendelian Randomization (MR) to investigate causal relationships, the result identified strong evidence that the trait of Neuroticism is a causal risk factor for MDD. A reduction of the Neuroticism score by 4 points reduces the chance of MDD by about 25.0% [30]. Other evidence indirectly favoring the causal relationship between personality traits and MDD may be that even after remission from MDD, personality traits tended to be well preserved [31, 32]. Therefore, assessment of personality traits might help identify medical students who are potentially more prone to develop MDD in the future and can be prevented by providing early preventative interventions.

Personality traits were linked to coping mechanisms one used when facing life challenges or stressors. Neuroticism was highly related to escape-avoidant coping, Agreeableness was negatively related to confronted coping, and Conscientiousness was related to problem-solving and negatively related to escape avoidance. Additionally, a lower score of Conscientiousness and a higher score of Neuroticism were associated with the presence of residual depressive symptoms in treated individuals with MDD [18]. This information may help us understand some individuals with MDD/dysthymia in our study who still had depressive symptoms despite receiving adequate pharmacological treatment. Apart from pointing out the population at risk for developing MDD, knowing personality traits can guide us to the underlying coping mechanism and target improving the immature coping mechanism for better treatment outcomes. For the Faculty of Medicine policy, establishing a personality traits assessment may assist in the medical student entrance examination process to identify who is more prone to develop MDD. Additionally, providing early preventative intervention programs to make medical students aware of their personality traits or coping mechanisms may help them find more adaptive coping mechanisms and areas for self-development. These processes may reduce the chance of MDD among them in the future.

The study of personality traits in Thailand was still limited. In Thailand, this was the first one which had conducted for medical students. We tried to enroll subjects from all regions of Thailand, but by convenience collecting process, the sample size had no equal probability of providing a sample in each region. The population was dominated by the Southern institutions. The response rate was 70.9% of medical students who were members of medical student's official social media. So, this dataset might not fairly represent Thai medical students countrywide. Due to its cross-sectional design, the result could only provide association but not cause-and-effect interpretation and was limited in its ability to infer causality. Therefore, future longitudinal research to explore causality would be valuable. Another limitation was the use of self-administered questionnaires which can be misunderstood regarding the intended meaning of the questions. We minimized this problem by choosing the questionnaires with good reliability or good Cronbach’s alpha coefficient. However, the notably low Cronbach's alpha of 0.37 for the Agreeableness domain suggested that this domain might not be reliably assessed with the IPIP-NEO Thai version. This could affect the interpretation of results related to the Agreeableness domain. However, for the data in this study, Cronbach's alpha coefficient of 0.79 for the Agreeableness domain suggested that this tool is reasonably reliable. In addition, the online questionnaire method was efficient and could reach a wide audience but might introduce bias, as it might exclude those without internet access or those less inclined to participate in online surveys or include those more have a particular interest in mental health. Therefore, future studies could address these limitations, perhaps through stratified sampling or more multi-center collaboration, which would be valuable.

Further studies in this field should be conducted in a longitudinal design to investigate the temporal relationship between personality traits and MDD. Other psychiatric diagnoses are also interesting to be explored about personality traits. The coping mechanism is the other issue concerning personality traits. In addition, cultural factors might influence the expression of personality traits and their association with mental health outcomes. So, a deeper examination of how cultural factors affected them, may need further understanding.

## Conclusion

More than a quarter of Thai medical students reported MDD. A higher score in Neuroticism and a lower score in Extraversion, Agreeableness, and Conscientiousness related to MDD. Therefore, medical schools may benefit from knowing medical students’ personality traits, to identify coping mechanisms and predict those at a higher risk of developing MDD in the future.

### Supplementary Information

**Supplementary Material 1. **

## Data Availability

The qualitative data of the current study cannot be made publicly available for confidentiality reasons, but they can be available on request by the corresponding author.

## Abbreviations

IPIP-NEO: International Personality Item Pool-NEO
MDD: Major depressive disorder
PHQ-9: Patient Health Questionnaire-9
