# Supplementary material for: The personality traits with depression and suicidal ideation among Thai medical students: a university-based multiregional study
Source: BMC Psychol. 2024 Apr 23;12:223. doi: 10.1186/s40359-024-01707-8 (PMC11040884; doi:10.1186/s40359-024-01707-8)
Supplement: Supplementary file 1 — Supplementary Material 1. [file 40359_2024_1707_MOESM1_ESM.pdf]

แบบทดสอบภาวะซึมเศร้า

คำชี้แจง: ในช่วง 2 สัปดาห์ ที่ผ่านมา ท่านมีอาการดังต่อไปนี้บ่อยแค่ไหน (ทำเครื่องหมาย √ ในช่องที่ตรงคำตอบของท่าน)

**คำถาม**	**อาการในช่วง 2 สัปดาห์ที่ผ่านมา**			
	**0**	**1**	**2**	**3**
	ไม่เลย	มีบางวัน<br>ไม่บ่อย	มีค่อนข้าง<br>บ่อย	มีเกือบ<br>ทุกวัน
1. เบื่อ ทำอะไรๆ ก็ไม่เพลิดเพลิน				
2. ไม่สบายใจ ซึมเศร้า หรือท้อแท้				
3. หลับยาก หรือหลับๆ ตื่นๆ หรือหลับมากเกินไป				
4. เหนื่อยง่าย หรือไม่ค่อยมีแรง				
5. เบื่ออาหาร หรือกินมากเกินไป				
6. รู้สึกไม่ดีกับตัวเอง คิดว่าตัวเองล้มเหลว หรือเป็นคนทำให้ตัวเองหรือครอบครัวผิดหวัง				
7. สมาธิไม่ดีเวลาทำอะไร เช่น ดูโทรทัศน์ ฟังวิทยุ หรือทำงานที่ต้องใช้ความตั้งใจ				
8. พูดหรือทำอะไรช้าจนคนอื่นมองเห็นหรือกระสับกระส่ายจนท่านอยู่ไม่นิ่งเหมือนเคย				
9. คิดทำร้ายตนเอง หรือคิดว่าถ้าตายไปเสียคงจะดี				

แบบทดสอบบุคลิกภาพ

คำชี้แจง: ท่านมีความคิดเห็นว่าบุคลิกภาพของท่านตรงกับคำบรรยายในแต่ละข้อเพียงใด (ทำเครื่องหมาย √ ในช่องที่ตรงคำตอบของท่าน)

บุคลิกภาพ	ความคิดเห็น				
	1	2	3	4	5
	เห็นด้วยน้อยที่สุด	เห็นด้วยน้อย	เห็นด้วยปานกลาง	เห็นด้วยมาก	เห็นด้วยอย่างยิ่ง
1. ฉันรู้สึกหม่นหมองอยู่บ่อยๆ					
2. ฉันเชื่อในความสำคัญของศิลปะ					
3. ฉันไม่ค่อยรู้สึกหงุดหงิด					
4. ฉันพูดน้อย					
5. ฉันปล่อยเวลาให้ผ่านไปโดยเปล่าประโยชน์					
6. ฉันเชื่อว่าผู้อื่นมีเจตนาและความตั้งใจดี					
7. ฉันเป็นเพื่อนกับคนอื่นง่าย					
8. ฉันใส่ใจกับรายละเอียด					
9. ฉันมีแนวโน้มที่จะลงคะแนนให้กับนักการเมืองที่มีความเป็นเสรีนิยม					
10. ฉันไม่ชอบตัวของฉันเอง					
11. ฉันไม่ค่อยพูด					
12. ฉันหลีกเลี่ยงที่จะทำงานตามความรับผิดชอบของฉัน					
13. ฉันมักรู้สึกหดหู่					
14. ฉันเคารพผู้อื่น					
15. ฉันทำให้ผู้อื่นรู้สึกผ่อนคลายและสบายใจ					
16. ฉันยอมรับผู้อื่นในแบบที่เขาเป็น					
17. อารมณ์ของฉันเปลี่ยนแปลงบ่อย					
18. ฉันจัดการงานหรือความยุ่งยากต่างๆ ให้เสร็จได้อย่างรวดเร็ว					
19. ฉันพอใจในตัวเองเป็นอย่างมาก					

**บุคลิกภาพ**	**ความคิดเห็น**				
	**1**	**2**	**3**	**4**	**5**
	เห็นด้วยน้อยที่สุด	เห็นด้วยน้อย	เห็นด้วยปานกลาง	เห็นด้วยมาก	เห็นด้วยอย่างยิ่ง
20. ฉันทำตามแผนการของฉัน					
21. ฉันไม่ชอบศิลปะ					
22. ฉันทำงานเพียงแค่ให้มันเสร็จๆ ไป					
23. ฉันมักวางแผนและทำตามแผนการที่วางไว้อย่างเคร่งครัด					
24. ฉันไม่ค่อยรู้สึกหดหู่					
25. ฉันหลีกเลี่ยงการพูดคุยเรื่องเชิงปรัชญา					
26. ฉันรู้สึกสบายใจกับตัวฉันเอง					
27. ฉันไม่รู้สึกสนุกกับการไปพิพิธภัณฑ์ศิลปะ					
28. ฉันพยายามไม่ทำตัวให้เป็นจุดเด่นหรือที่สนใจของผู้อื่น					
29. ฉันมีแนวโน้มที่จะลงคะแนนให้นักการเมืองที่มีความเป็นอนุรักษ์นิยม					
30. ฉันรู้สึกว่ามันยากที่จะเริ่มต้นทำงานหรือทำอะไรบางอย่างอย่างตั้งใจ					
